# *meso*-1-{[2-(Propyl-1-sulfin­yl)eth­yl]sulfin­yl}propane

**DOI:** 10.1107/S1600536810005799

**Published:** 2010-02-17

**Authors:** Solange M. S. V. Wardell, James L. Wardell, Geraldo M. de Lima, Edward R. T. Tiekink

**Affiliations:** a CHEMSOL, 1 Harcourt Road, Aberdeen AB15 5NY, Scotland; b Centro de Desenvolvimento Tecnológico em Saúde (CDTS), Fundação Oswaldo Cruz (FIOCRUZ), Casa Amarela, Campus de Manguinhos, Av. Brasil 4365, 21040-900, Rio de Janeiro, RJ, Brazil; c Departamento de Quimica, ICEx, Universidade Federal de Minas Gerais, 31270-901 Belo Horizonte, MG, Brazil; d Department of Chemistry, University of Malaya, 50603 Kuala Lumpur, Malaysia

## Abstract

The title mol­ecule, C_8_H_18_O_2_S_2_, is disposed about a centre of inversion implying an *anti*-disposition of the sulfinyl-O atoms; the terminal *n*-propyl group has an extended conformation. The crystal packing is dominated by C—H⋯O inter­actions, which lead to the formation of supra­molecular arrays in the *bc* plane.

## Related literature

For the structures of the stereoisomers of *RS*(=O)CH_2_CH_2_S(=O)*R*, see: Pelizzi *et al.* (1976 ▶); Svinning *et al.* (1976 ▶); Chu & Madden (1978 ▶); Ternay *et al.* (1978 ▶); Cattalini *et al.* (1979 ▶); Li *et al.* (2002 ▶, 2004 ▶). For the preparation and separation of the steroisomers of the title compound, see: Hull & Bargar (1975 ▶); Li *et al.* (2005 ▶). For information on the use of bis-sulfoxides as a ligand, see: de Souza *et al.* (1995 ▶, 1997 ▶); Huang *et al.* (1986 ▶); Huang & Zhang (1986 ▶); Filgueiras & Marques (1985 ▶); Filgueiras *et al.* (1982 ▶); Bu *et al.* (2002 ▶); Li *et al.* (2005 ▶); Yapp *et al.* (1997 ▶).
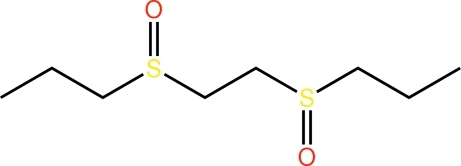


## Experimental

### 

#### Crystal data

C_8_H_18_O_2_S_2_
*M* *_r_* = 210.34
Monoclinic, 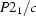

*a* = 11.9794 (9) Å
*b* = 5.2190 (3) Å
*c* = 8.7618 (5) Å
β = 97.191 (5)°
*V* = 543.48 (6) Å^3^
*Z* = 2
Mo *K*α radiation
μ = 0.45 mm^−1^
*T* = 120 K
1.1 × 0.6 × 0.12 mm

#### Data collection

Nonius KappaCCD area-detector diffractometer
Absorption correction: multi-scan (*SADABS*; Sheldrick, 2007 ▶) *T*_min_ = 0.527, *T*_max_ = 0.746
5666 measured reflections
1239 independent reflections
1167 reflections with *I* > 2σ(*I*)
*R*_int_ = 0.042

#### Refinement

*R*[*F* ^2^ > 2σ(*F* ^2^)] = 0.031
*wR*(*F* ^2^) = 0.080
*S* = 1.06
1239 reflections
56 parameters
H-atom parameters constrained
Δρ_max_ = 0.33 e Å^−3^
Δρ_min_ = −0.35 e Å^−3^

### 

Data collection: *COLLECT* (Hooft, 1998 ▶); cell refinement: *DENZO* (Otwinowski & Minor, 1997 ▶) and *COLLECT*; data reduction: *DENZO* and *COLLECT*; program(s) used to solve structure: *SHELXS97* (Sheldrick, 2008 ▶); program(s) used to refine structure: *SHELXL97* (Sheldrick, 2008 ▶); molecular graphics: *ORTEP-3* (Farrugia, 1997 ▶) and *DIAMOND* (Brandenburg, 2006 ▶); software used to prepare material for publication: *publCIF* (Westrip, 2010 ▶).

## Supplementary Material

Crystal structure: contains datablocks general, I. DOI: 10.1107/S1600536810005799/ez2201sup1.cif

Structure factors: contains datablocks I. DOI: 10.1107/S1600536810005799/ez2201Isup2.hkl

Additional supplementary materials:  crystallographic information; 3D view; checkCIF report

## Figures and Tables

**Table 1:** Hydrogen-bond geometry (Å, °)

*D*—H⋯*A*	*D*—H	H⋯*A*	*D*⋯*A*	*D*—H⋯*A*
C1—H1b⋯O1^i^	0.99	2.41	3.3035 (19)	150
C4—H4a⋯O1^i^	0.99	2.40	3.2751 (19)	147
C4—H4b⋯O1^ii^	0.99	2.57	3.5124 (18)	159

Symmetry codes: (i) 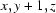; (ii) 
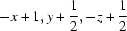
.

**Fig. 1.**
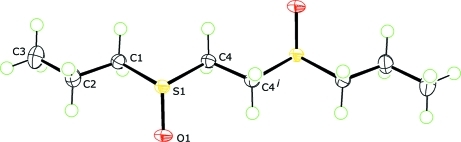
The molecular structure of (I) showing the atom-labelling scheme and displacement ellipsoids at the 70% probability level.

**Fig. 2.**
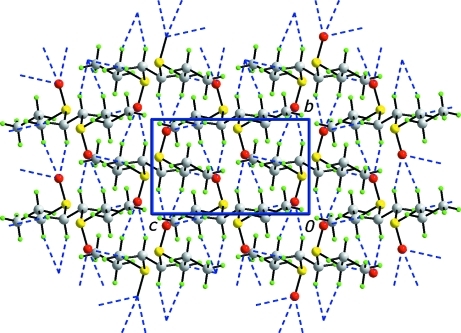
A view of a supramolecular array in (I) in the bc plane. The C–H···O interactions are shown as blue dashed lines. Colour code: S, yellow; O, red; C, grey; and H, green.

**Fig. 3.**
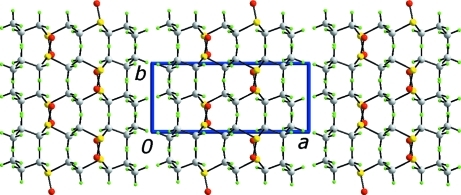
View in projection down the c axis of the unit cell contents in (I). Colour code: S, yellow; O, red; C, grey; and H, green.

## supplementary crystallographic information

### Comment

Bis-sulfoxides such as the title compound, (I), have found use as ligands (de Souza *et al.*, 1995, 1997; Huang *et al.*, 1986; Huang & Zhang, 1986; Filgueiras & Marques, 1985; Filgueiras *et al.*, 1982; Bu *et al.*, 2002; Li *et al.*, 2005; Yapp *et al.*, 1997). Details on the preparation and the separation of steroisomers of (I) are available in the literature (Hull & Bargar 1975; Li *et al.* 2005). Crystallography shows the molecule of (I) is disposed about a centre of inversion, Fig. 1, and the n-propyl chain has an extended conformation as seen in the value of the S1–C1–C2–C3 torsion angle of S1–C1–C2–C3 of -179.93 (11) °. The *anti*-disposition of the sulfinyl-O atoms allow for the optimisation of C–H···O interactions in the crystal structure, Table 1. Thus, each sulfinyl-O1 associates with three methylene-H atoms to form a supramolecular array in the *bc* plane, Fig. 2, which stack along the *a* axis, Fig. 3.

The central core in (I), including its disposition about a centre of inversion, resembles that found in each of the reported *meso*-RS(═ O)CH_2_CH_2_S(═O)R structures, where R = Me (Svinning *et al.*, 1976), Et (Li *et al.*, 2004), Ph (Pelizzi, *et al.* 1976; Ternay *et al.* 1978; Cattalini *et al.*, 1979), benzyl (Li *et al.* 2002), and mesityl (Chu & Madden, 1978), but not in their homo chiral stereoisomers where R = Ph (Ternay *et al.* 1978; Cattalini *et al.*, 1979) and mesityl (Chu & Madden, 1978).

### Experimental

Compound (I) was prepared by a published method (Li *et al.*, 2005) and had spectral and other parameters in agreement with published values (Li *et al.*, 2005; Yapp *et al.*, 1997). M.pt. 434-435 K. Lit. value 434-435 K (Hull & Bargar, 1975; Li *et al.*, 2005). The sample used in the crystallographic study was grown from an ethanol solution of (I).

### Refinement

The C-bound H atoms were geometrically placed (C–H = 0.98–0.99 Å) and refined as riding with *U_iso_*(H) = 1.2-1.5*U_eq_*(C).

### Crystal data

**Table:**

C_8_H_18_O_2_S_2_	*F*(000) = 228
*M**_r_* = 210.34	*D*_x_ = 1.285 Mg m^−^^3^
Monoclinic, *P*2_1_/*c*	Mo *K*α radiation, λ = 0.71073 Å
Hall symbol: -P 2ybc	Cell parameters from 19160 reflections
*a* = 11.9794 (9) Å	θ = 2.9–27.5°
*b* = 5.2190 (3) Å	µ = 0.45 mm^−^^1^
*c* = 8.7618 (5) Å	*T* = 120 K
β = 97.191 (5)°	Plate, colourless
*V* = 543.48 (6) Å^3^	1.1 × 0.6 × 0.12 mm
*Z* = 2	

### Data collection

**Table:**

Nonius KappaCCD area-detector diffractometer	1239 independent reflections
Radiation source: Enraf Nonius FR591 rotating anode	1167 reflections with *I* > 2σ(*I*)
10 cm confocal mirrors	*R*_int_ = 0.042
Detector resolution: 9.091 pixels mm^-1^	θ_max_ = 27.5°, θ_min_ = 3.4°
φ and ω scans	*h* = −12→15
Absorption correction: multi-scan (*SADABS*; Sheldrick, 2007)	*k* = −6→6
*T*_min_ = 0.527, *T*_max_ = 0.746	*l* = −11→11
5666 measured reflections	

### Refinement

**Table:**

Refinement on *F*^2^	Primary atom site location: structure-invariant direct methods
Least-squares matrix: full	Secondary atom site location: difference Fourier map
*R*[*F*^2^ > 2σ(*F*^2^)] = 0.031	Hydrogen site location: inferred from neighbouring sites
*wR*(*F*^2^) = 0.080	H-atom parameters constrained
*S* = 1.06	*w* = 1/[σ^2^(*F*_o_^2^) + (0.0344*P*)^2^ + 0.4193*P*] where *P* = (*F*_o_^2^ + 2*F*_c_^2^)/3
1239 reflections	(Δ/σ)_max_ = 0.001
56 parameters	Δρ_max_ = 0.33 e Å^−^^3^
0 restraints	Δρ_min_ = −0.35 e Å^−^^3^

### Special details

**Table:**

Geometry. All s.u.'s (except the s.u. in the dihedral angle between two l.s. planes) are estimated using the full covariance matrix. The cell s.u.'s are taken into account individually in the estimation of s.u.'s in distances, angles and torsion angles; correlations between s.u.'s in cell parameters are only used when they are defined by crystal symmetry. An approximate (isotropic) treatment of cell s.u.'s is used for estimating s.u.'s involving l.s. planes.
Refinement. Refinement of *F*^2^ against ALL reflections. The weighted *R*-factor wR and goodness of fit *S* are based on *F*^2^, conventional *R*-factors *R* are based on *F*, with *F* set to zero for negative *F*^2^. The threshold expression of *F*^2^ > 2σ(*F*^2^) is used only for calculating *R*-factors(gt) etc. and is not relevant to the choice of reflections for refinement. *R*-factors based on *F*^2^ are statistically about twice as large as those based on *F*, and *R*- factors based on ALL data will be even larger.

### Fractional atomic coordinates and isotropic or equivalent isotropic displacement parameters (Å^2^)

**Table:**

	*x*	*y*	*z*	*U*_iso_*/*U*_eq_	
S1	0.66819 (3)	−0.09909 (6)	0.45405 (4)	0.01305 (14)	
O1	0.64458 (10)	−0.37397 (19)	0.40786 (13)	0.0187 (3)	
C1	0.72083 (12)	0.0560 (3)	0.29375 (16)	0.0149 (3)	
H1A	0.6705	0.0192	0.1978	0.018*	
H1B	0.7228	0.2438	0.3097	0.018*	
C2	0.83896 (12)	−0.0425 (3)	0.27975 (17)	0.0191 (3)	
H2A	0.8363	−0.2305	0.2646	0.023*	
H2B	0.8885	−0.0065	0.3765	0.023*	
C3	0.88818 (14)	0.0828 (3)	0.14553 (19)	0.0246 (4)	
H3A	0.8398	0.0455	0.0494	0.037*	
H3B	0.9638	0.0147	0.1397	0.037*	
H3C	0.8925	0.2686	0.1614	0.037*	
C4	0.53341 (12)	0.0583 (3)	0.44006 (15)	0.0144 (3)	
H4A	0.5438	0.2443	0.4589	0.017*	
H4B	0.4924	0.0343	0.3358	0.017*	

### Atomic displacement parameters (Å^2^)

**Table:**

	*U*^11^	*U*^22^	*U*^33^	*U*^12^	*U*^13^	*U*^23^
S1	0.0150 (2)	0.0110 (2)	0.0135 (2)	0.00060 (11)	0.00307 (13)	0.00048 (11)
O1	0.0238 (6)	0.0087 (5)	0.0244 (6)	0.0012 (4)	0.0066 (4)	0.0004 (4)
C1	0.0176 (7)	0.0133 (6)	0.0147 (6)	−0.0002 (5)	0.0048 (5)	0.0007 (5)
C2	0.0179 (7)	0.0221 (7)	0.0181 (7)	0.0014 (6)	0.0057 (5)	0.0005 (6)
C3	0.0210 (8)	0.0325 (9)	0.0217 (7)	−0.0031 (6)	0.0078 (6)	0.0010 (6)
C4	0.0167 (7)	0.0111 (6)	0.0162 (6)	0.0018 (5)	0.0049 (5)	0.0013 (5)

### Geometric parameters (Å, °)

**Table:**

S1—O1	1.5077 (10)	C2—H2B	0.9900
S1—C4	1.8018 (14)	C3—H3A	0.9800
S1—C1	1.8021 (14)	C3—H3B	0.9800
C1—C2	1.525 (2)	C3—H3C	0.9800
C1—H1A	0.9900	C4—C4^i^	1.525 (3)
C1—H1B	0.9900	C4—H4A	0.9900
C2—C3	1.527 (2)	C4—H4B	0.9900
C2—H2A	0.9900		
			
O1—S1—C4	106.20 (7)	H2A—C2—H2B	107.9
O1—S1—C1	106.88 (7)	C2—C3—H3A	109.5
C4—S1—C1	98.06 (6)	C2—C3—H3B	109.5
C2—C1—S1	109.27 (10)	H3A—C3—H3B	109.5
C2—C1—H1A	109.8	C2—C3—H3C	109.5
S1—C1—H1A	109.8	H3A—C3—H3C	109.5
C2—C1—H1B	109.8	H3B—C3—H3C	109.5
S1—C1—H1B	109.8	C4^i^—C4—S1	108.34 (13)
H1A—C1—H1B	108.3	C4^i^—C4—H4A	110.0
C1—C2—C3	111.67 (13)	S1—C4—H4A	110.0
C1—C2—H2A	109.3	C4^i^—C4—H4B	110.0
C3—C2—H2A	109.3	S1—C4—H4B	110.0
C1—C2—H2B	109.3	H4A—C4—H4B	108.4
C3—C2—H2B	109.3		
			
O1—S1—C1—C2	−71.04 (11)	O1—S1—C4—C4^i^	65.40 (14)
C4—S1—C1—C2	179.23 (10)	C1—S1—C4—C4^i^	175.68 (13)
S1—C1—C2—C3	−179.93 (11)		

Symmetry codes: (i) −*x*+1, −*y*, −*z*+1.

### Hydrogen-bond geometry (Å, °)

**Table:**

*D*—H···*A*	*D*—H	H···*A*	*D*···*A*	*D*—H···*A*
C1—H1b···O1^ii^	0.99	2.41	3.3035 (19)	150
C4—H4a···O1^ii^	0.99	2.40	3.2751 (19)	147
C4—H4b···O1^iii^	0.99	2.57	3.5124 (18)	159

Symmetry codes: (ii) *x*, *y*+1, *z*; (iii) −*x*+1, *y*+1/2, −*z*+1/2.
